# Optimal Information Transfer in the Cortex through Synchronization

**DOI:** 10.1371/journal.pcbi.1000934

**Published:** 2010-09-16

**Authors:** Andres Buehlmann, Gustavo Deco

**Affiliations:** 1 Computational Neuroscience, Universitat Pompeu Fabra, Barcelona, Spain; 2 Institució Catalana d'Estudis Avançats (ICREA), Barcelona, Spain; University College London, United Kingdom

## Abstract

In recent experimental work it has been shown that neuronal interactions are modulated by neuronal synchronization and that this modulation depends on phase shifts in neuronal oscillations. This result suggests that connections in a network can be shaped through synchronization. Here, we test and expand this hypothesis using a model network. We use transfer entropy, an information theoretical measure, to quantify the exchanged information. We show that transferred information depends on the phase relation of the signal, that the amount of exchanged information increases as a function of oscillations in the signal and that the speed of the information transfer increases as a function of synchronization. This implies that synchronization makes information transport more efficient. In summary, our results reinforce the hypothesis that synchronization modulates neuronal interactions and provide further evidence that gamma band synchronization has behavioral relevance.

## Author Summary

Different brain areas are involved in any cognitive task. This implies that information has to be transmitted between different brain areas. Recent experimental results suggest that synchronization plays a crucial role in information exchange between cortical areas. They show that synchronization is capable of rendering network connections effective or ineffective. We study this hypothesis using a neurodynamical model and present results suggesting that both phase and strength of neuronal oscillations in the gamma frequency band influence amount and speed of information transport. We conclude that neuronal synchronization is crucial for information transmission and therefore might even have behavioral relevance.

## Introduction

Gamma band synchronization has been found in many cortical areas and in a variety of tasks. It has been studied most extensively in the visual cortex of cats and monkeys [1]–[8]. Several authors have proposed that these synchronizations influence the interactions among neuronal groups [9], [10], a hypothesis referred to as communication through coherence (CTC, [11]). In computational studies, it has been shown that entrainment enhances transmitted information between input and output spikes [12], that synchronization in the gamma frequency range increases the effective synaptic gain for the responses to an attended stimulus [13] and that the transmission time of responses of coupled oscillators depends on the phase difference in the stable synchronized state [14]. Also, several experimental studies have presented results supporting the CTC hypothesis [15]–[19].

In this study, we concentrate on the results shown by Womelsdorf et al. [17]. They explore the mutual influence of two groups of neurons as a function of their phase shift. These authors quantify the mutual influence of the multi unit activity (MUA) in the two groups as the Spearman rank correlation coefficient of the two MUA's 60 Hz power. They show evidence that the correlation between the two groups of neurons varies as a function of the phase shift of the oscillations at 60 Hz. There is a specific phase shift at which the correlation between the two groups is highest. [17] conclude that the effective connectivity in a network can thus be maximized or minimized through synchronization of a specific phase relation, resulting in an effective interaction pattern.

While the results presented by [17] clearly support the CTC hypothesis, they leave some open questions. Is it only the 60 Hz power that depends on the 60 Hz phase? Do the MUAs only correlate or is there mutual interaction between the two? Is this effect restricted to the gamma band or can it be generalized to other frequency bands? What is the influence of the total gamma power in the signal?

Here, to address these questions, we use a detailed biophysical model network with realistic spiking properties. A first advantage of using a model is that we can generate more data than in an experiment. This makes it possible to use an information theoretical measure for the mutual interaction instead of rank correlation. Many different interdependence measures such as mutual information, transfer information, nonlinear regression, phase synchronization and generalized synchronization have recently been proposed (see [20] and [21] for comparisons of the different methods). It has become evident that the appropriateness of each measure is determined by the data it is applied to. Thus, given our current data set, we opted to use transfer entropy (TE), introduced by [22]. The TE is an information theoretical measure that quantifies the statistical coherence between systems. It has the advantage that it does not only measure the coherence between two signals, but is able to distinguish between driving and responding elements and therefore between shared and transported information. This is called the directionality of the information flow. We measure the TE between the MUA of the two neuronal clusters, which allows us to study the interdependence of the spiking activity in each of them and not just the correlation of the spectral power in a specific frequency band, as was done in the experimental work. A further crucial advantage of the model is that we can change network parameters systematically and explore the dynamical range of the network.

The model we use in this study consists of integrate-and-fire neurons. One of two pools of excitatory neurons receives input (Poisson spike train) which it passes to a neighboring pool, connected by feedforward and feedback connections. Each pool of excitatory neurons is connected to a pool of inhibitory neurons, which generates oscillations in the gamma frequency band through a pyramidal-interneuron feedback loop [23]. Beta oscillations are obtained from the same network by parameter modification. Several methods have been proposed to generate beta oscillations [24], [25]. Here, for the sake of simplicity, we modify the decay constants of the synapses. We show that the correlation as measured by the Spearman rank correlation coefficient depends on the phase relation in the gamma band. This result confirms the experimental finding of [17]. Secondly, after applying TE to measure the information exchange between two pools, we find that TE very similarly depends on the phase shift, i.e., that there is an optimal phase relation where the TE is maximal. Thirdly, we reveal such dependence also in the beta band. Fourthly, we demonstrate that the TE increases as a function of the power in the gamma band. Lastly, we show that the information exchange gets faster if the gamma band synchronization increases. In sum, we provide support for the CTC hypothesis and make the prediction that CTC is a general mechanism, not restricted to the gamma band.

## Methods

### Experimental analysis

**Figure 1:**
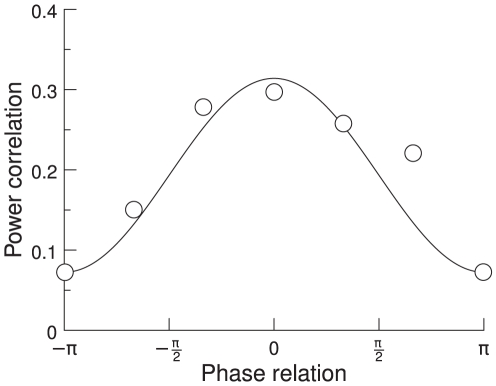
Spearman rank correlation coefficient. The rank correlation coefficient between the two MUAs' 60 Hz power is plotted as a function of their phase relation. The solid line indicates a cosine fit. Adapted from [17].

Womelsdorf et al. [17] analyzed four different data sets. The first data set consisted of measures from awake cats in area 17 [26], the second from awake cats in areas 18 and 21a [4], the third from awake monkeys in area V1 and the fourth from awake monkeys in area V4 [3], [7]. In all four data sets they recorded multi unit activity simultaneously from 4 to 8 electrodes. For each pair of neuronal groups, they quantified the synchronization by MUA-MUA phase coherence spectrum, which showed a peak in the gamma frequency band. These authors then calculated the Spearman rank correlation coefficient between the two MUAs' 60 Hz power. They found that the fluctuations of the 60 Hz power were most strongly correlated when the 60 Hz phase relation was close to its mean, as illustrated in Fig. 1. From this they concluded that effective connectivity can be maximized or minimized through synchronization at a favorable or unfavorable phase relation.

### Theoretical framework

We use a model with leaky integrate-and-fire (IF) dynamics, following [27]. Each IF unit charges up to its stationary value as long as its membrane potential stays below a threshold. The membrane potential 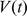 is given by:
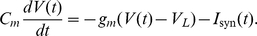
(1)

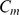
 is a membrane capacitance, 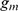 a membrane leak conductance, 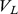 a resting potential and 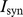 is the total synaptic current flowing into the cell. When the membrane potential reaches the threshold potential, it sends out a spike to all connected neurons and resets its membrane potential to the reset potential. The circuit remains shunted for a refractory period. Synaptic currents are mediated by excitatory (AMPA and NMDA) and inhibitory (GABA) receptors. The total synaptic current is given by
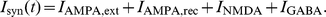
(2)


The currents are defined as follows:
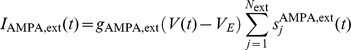
(3)

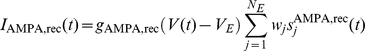
(4)

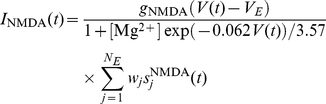
(5)

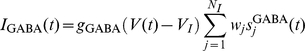
(6)

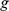
 denotes the receptor specific synaptic conductances, 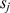 the fractions of open channels and the 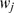 the synaptic weights. 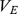 and 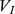 are the reversal potentials of the excitatory and inhibitory neurons, respectively, 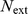 is the number of neurons encoding the spontaneous activity in the cortex, and 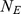 and 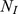 are the numbers of excitatory and inhibitory neurons in the network. The sum in each expression runs over all neurons, summing their open channels, weighted by the synaptic weights that implement the connection strengths between neurons. The NMDA synaptic current is dependent on the membrane potential and controlled by the extracellular concentration of 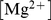.

The fractions of open channels are given by:
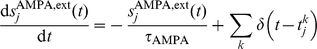
(7)

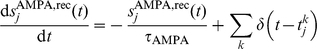
(8)

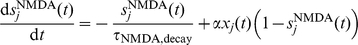
(9)

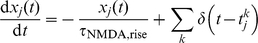
(10)

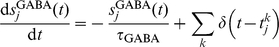
(11)

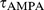
, 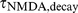 and 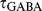 are the decay times and 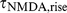 is the rise time for the corresponding synapses. AMPA has a very short decay time (2 ms) while NMDA has a long one (100 ms) and the GABA decay time lies in-between (10 ms). The rise times of AMPA and GABA currents are neglected, as they are typically very short (<1 ms). The sums over 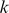 represent a sum over spikes formulated as 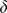-peaks (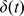) emitted by presynaptic neuron 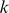 at time 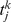. All input is generated via a Poisson process.

The equations are integrated using a fourth order Runge-Kutta method with a time step of 0.02 ms. The network is organized in pools. Neurons within a specific pool have stronger recurrent connections than neurons between the pools. The intention of this work is to study cortical neural interactions not limited to a specific brain area. However, as our simulations needed to be directly comparable to [17], and have specific parameter sets, our network models two clusters of cortical neurons in visual cortex V4.

**Figure 2:**
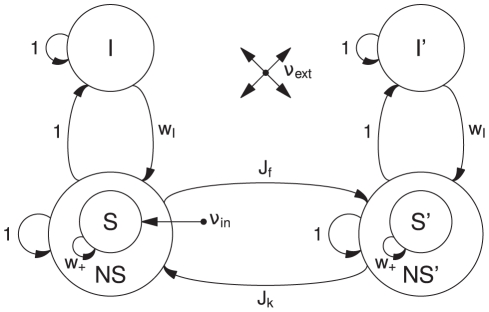
Schematic representation of the network. The network consists of two parts. In each part, there are excitatory (S, NS) and inhibitory (I) neurons. The excitatory neurons are divided into two pools. The selective pool (S) receives the external input (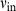) and has strong recurrent connections (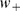). The non-selective pool (NS) simulates the surrounding cerebral cortex. In each part of the network, the excitatory neurons are connected to a pool of inhibitory neurons (I) via connection weights 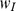. The two parts of the network are connected via the selective pools. There are both feedforward (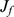) and feedback (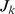) connections. The network is exposed to an external current 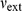, modeling the spontaneous activity observed in the cerebral cortex.

The network model consists of two parts (Fig. 2). In each part there are pools of excitatory and inhibitory neurons, with a total of 800 excitatory and 200 inhibitory neurons. The excitatory neurons are subdivided into a selective pool and a non-selective pool. The neurons in the selective pools (S,S′) are the ones that receive input either from outside or from the connected selective pool. The non-selective neurons (NS, NS′) simulate the surrounding brain areas. Each population of excitatory neurons is connected to a pool of inhibitory neurons (I, I′). This allows for generating oscillations in each population separately. The two parts of the network are connected via feedforward (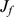) and feedback (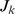) connections that project onto the selective pools. The external input (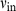) is a Poisson spike train that projects to the selective pool (S) of the first part of the network. In addition to the recurrent connections, the network is exposed to an external current (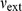), modeled as a Poisson spike train of 800 neurons, firing at 3 Hz. This models the spontaneous activity observed in the cerebral cortex. The network is fully connected.

Gamma oscillations in a network with excitatory and inhibitory neurons are generated through a pyramidal-interneuron feedback loop [23], [28]. Pyramidal neurons excite interneurons and interneurons in turn send inhibition back on pyramidal cells. The population frequency is determined by the sum of excitatory and inhibitory lags. The recurrent excitatory connections tend to decrease the oscillation frequency (as compared to only excitatory-inhibitory and inhibitory-excitatory connections) as they tend to prolong the positive phase in each cycle. In our network we can therefore generate and control the oscillations in the gamma frequency band by adjusting the AMPA and NMDA conductances. For example, increasing the 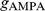 and decreasing 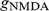 shifts the balance in the network towards fast excitation (AMPA) and slow inhibition (GABA) and thus increases the gamma frequency band oscillations. The conductances in our network are varied according to the following rule: 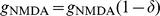 and 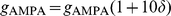. Throughout the paper, we will refer to the parameter 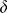 as the 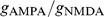 modification ratio. The factor 10 stems from the fact that near the firing threshold, the ratio of NMDA∶AMPA components becomes 10 in terms of charge entry, as stated in [27]. Therefore, in order not to change the spontaneous state, a decrease in 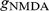 is compensated by a tenfold increase in 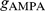. All recurrent conductances (both inhibitory and excitatory) are changed according to these rules.

By adjusting the synaptic decay constants, the oscillation frequency can be shifted into the beta band. The crucial parameter is 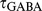. An increase of 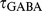 slows down the rhythm of the pyramidal-interneuronal loop and will therefore yield an oscillation at a lower frequency. To generate oscillations in the beta range (around 20 Hz), we use 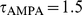 ms and 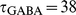 ms. To generate phase shifts in the gamma oscillations between the two parts of the network, we introduce a delay. The delay is set bidirectionally in the feedforward and feedback connections of the selective pools. Each spike emitted in 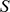 arrives at 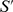 after 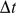 and vice versa. This lag in spike transmission generates a phase lag in the oscillations. A delay of, e.g., 4 ms yields a phase shift of about 90 in a 60 Hz oscillation. The actual value of the mean phase shift is not crucial to the obtained results. All trials are initiated with a period of 400 ms in which no stimulus is presented, followed by a period of 5500 ms composed of the presentation of the stimulus, followed by 100 ms in which no stimulus is present. Each simulation consists of 100 trials. All parameter values are listed in Table 1.

**Table 1:** The default parameter set.

Parameter	Value	Parameter	Value
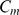 (excitatory)	0.5 nF	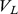	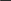 70 mV
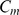 (inhibitory)	0.2 nF	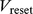	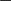 55 mV
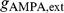 (excitatory)	2.08 nS	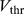	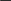 50 mV
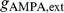 (inhibitory)	1.62 nS	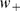	1.5
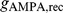 (excitatory)	0.104 nS	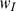	1.0
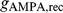 (inhibitory)	0.081 nS	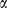	0.5 ms 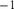
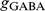 (excitatory)	1.287 nS	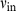	250 Hz
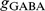 (inhibitory)	1.002 nS	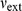	2.4 kHz
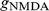 (excitatory)	0.327 nS	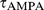	2 ms
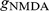 (inhibitory)	0.258 nS	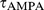 (beta osc.)	1.5 ms
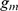 (excitatory)	25 nS	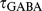	10 ms
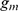 (inhibitory)	20 nS	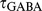 (beta osc.)	38 ms
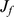	1.8	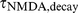	100 ms
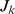	0.6	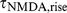	2 ms
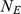	800	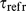 (excitatory)	2 ms
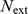	800	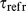 (inhibitory)	1 ms
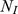	200	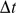 (delay)	4 ms
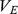	0 mV	feedback/feedforward ratio	1/3
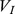	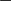 70 mV		

The default parameter set used in the integrate-and-fire simulations.

### Analysis

#### Multi unit activity

From our spiking simulations we calculate the multi unit activity (MUA) to analyze our simulations, in order to be able to compare our results directly with the experiments. To simulate the MUA, we randomly chose 10 neurons in each of the selective pools. This point process data is converted to a time series by binning the spikes in windows of 5 ms. The binning window is shifted in steps of 1 ms. The time series is then normalized to zero mean and unit variance. We use the normalized time series to estimate power spectrum and transfer entropy. Normalization is applied to rule out the possible influence of rate changes.

#### Power spectrum and phase estimation

We use the multitaper method [29], [30] to calculate the spectral power of our data. The signal in each time window (1000 ms) is multiplied with a set of Slepian data tapers. The tapered signal is then Fourier transformed, according to:
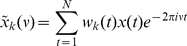
(12)where 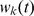 are 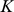 orthogonal taper functions, 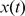 is the time series of our signal, and 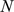 is the number of elements in each time window. The power spectrum is then the squared amplitude of 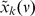, averaged over the 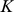 tapers. We used 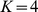 tapers in our study. The cross spectrum (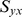) between two signals 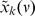 and 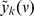 (averaged over 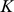 tapers) is given by
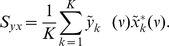
(13)The phase relation between two signals 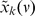 and 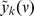 is defined as the angle of the cross spectrum. We use this method for phase estimation to be able to compare directly to the experimental results.

#### Transfer entropy

In Ref. [17] the mutual influence between two neuronal groups is quantified as the Spearman rank correlation coefficient of spectral power. The Spearman rank correlation is a non-parametric measure of correlation, which makes no assumptions about normality or linearity of the data. However, it is a symmetric measure and therefore fails to measure directionality of the flow of information. Thus, to overcome this limitation, here we use TE [22], which enables us to distinguish between shared and transported information. TE measures the deviation from the following generalized Markov property:
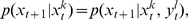
(14)where 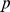 is the transition probability and 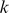 and 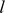 are the dimensions of the delay vectors. 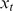 and 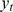 are the time series of the signal. We write 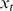 and 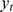 instead of 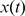 and 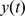, respectively, for better readability. If the deviation is small, then 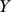 has no relevance for the transmission probability of 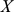. The incorrectness of this assumption can be quantified by the Kullback entropy
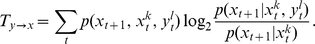
(15)In other words, transfer entropy represents the information about a future observation of variable 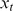 obtained from the simultaneous observation of past values of both 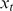 and 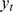, after discarding the information about the future of 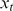 obtained from the past of 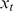 alone [20]. For computational reasons, we set 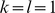. Conditional probabilities required in equation 15 are calculated from the joint probabilities. We approximate the joint probabilities by coarse-graining the continuous state space at resolution 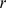 and using the histograms of the embedding vector (naive histogram technique [31]). When the available data is limited (number of samples 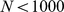) and the coupling between the time series is small, TE suffers from a finite sample effect, in particular for small resolution (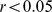), which makes the assessment of the significance of the obtained values difficult [31]. However, for all our simulations 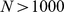 and 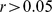, so we can assume that the finite sample issue affects our results to a negligible extent. We calculate the TE between the MUA in the two neuronal pools.

## Results

First we describe how the mean phase shift between pools of neurons is set by the delay in the feedforward and feedback connections. We then show that the correlation between the gamma power in the two pools depends on the phase relation in the gamma band. We demonstrate that TE has a very similar dependence on the phase shift and that TE increases as a function of gamma power. Finally, we reveal that if gamma power is high, information flow as measured by TE commences earlier.

### Delay–phase relation

**Figure 3:**
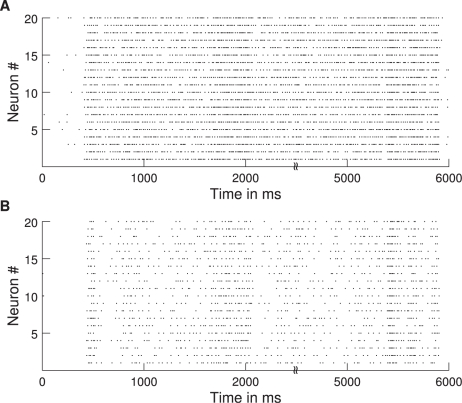
Raster plots. Raster plot of spikes of 20 neurons from the default simulations (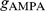/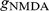 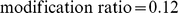). (**a**) Neurons from selective pool 1. (**b**) Neurons from selective pool 2.

**Figure 4:**
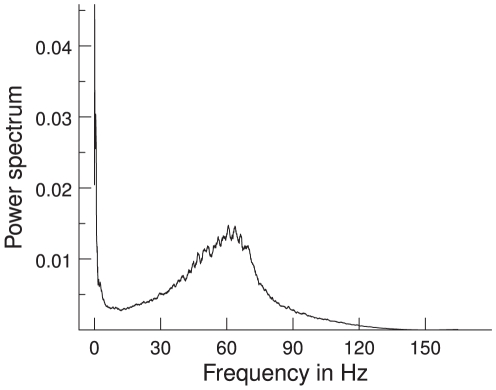
Power spectrum. The power spectrum of the MUA signal from a simulation with default parameters is shown. 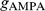/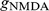 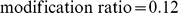, averaged over 100 trials.

**Figure 5:**
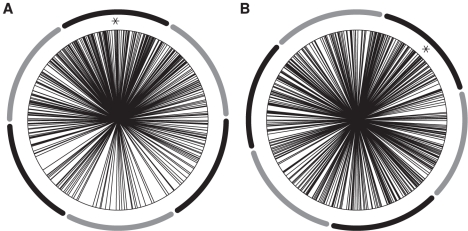
Phase distribution. The phases are widely distributed around the mean (marked with an asterix). The dark and light segments around the figures represent the phase bins into which trials were sorted. (**a**) Simulation: Phase distribution with an exemplary mean of 91.4 from the default simulations (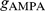/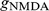 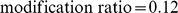). (**b**) Experiment: Phase distribution with a mean of 45.8. Adapted from [17].

Raster plots for 20 neurons from each neuronal pool are shown in (Fig. 3). The power spectrum of the MUA in our network shows a clear peak in the gamma band (Fig. 4), in accordance with the experimental results. Therefore, the introduced delay sets the phase shift for oscillations in the gamma band. The delay, however, sets only the mean phase shift, but the phase shifts fluctuate over time. Thus, even for a fixed delay they show a broad distribution around this mean phase shift. This distribution is shown in Fig. 5a. The mean phase in this specific simulation is 91.4°. This, however, is just an example, as the mean phase shift in the simulations can be set to any value by adjusting the delays accordingly. The phases are similarly widely distributed as in the experimental results by [17], shown in Fig. 5b.

### Dependence of correlations on phase

**Figure 6:**
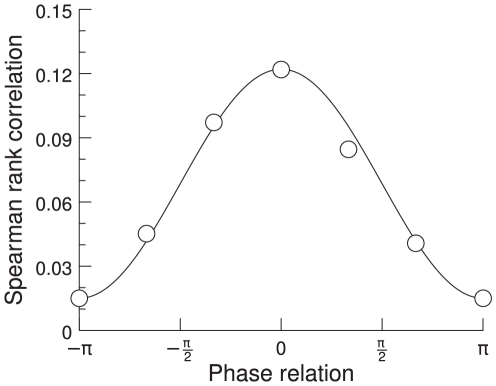
Spearman rank correlation coefficient. The rank correlation between the 60 Hz power in two neuronal pools is plotted as a function of the phase shift in the gamma band. A phase shift of zero represents the mean phase shift which is the point where the rank correlation is highest. The solid line indicates a cosine fit.

The phase shifts at 60 Hz between the two pools show a broad range of phases. We determine the phase shift in each time window of 500 ms. Then we calculate the correlation between the two pools for this time window by calculating the Spearman rank coefficient for the 60 Hz power in the two pools. The obtained correlation can now be sorted into different bins for the different phase shifts. We find that the correlation of the gamma band power between the two pools depends on the mean phase shift in the gamma band. Fig. 6 shows the rank correlation plotted against the phase shifts. The correlation is highest for the bin containing the mean phase shift and drops as it moves away from the mean. This confirms the experimental results of [17].

### Dependence of TE on phase

**Figure 7:**
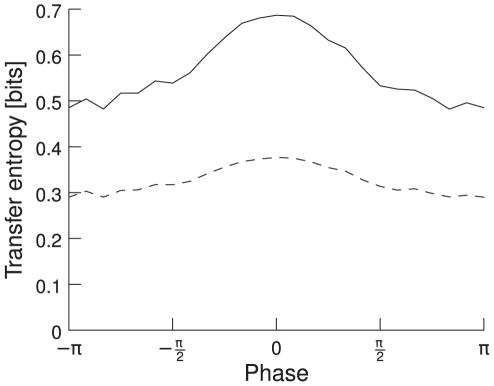
TE as a function of phase shifts and directionality. The phases are aligned relative to the mean phase, i.e., a phase shift of zero represents mean phase shift. TE is highest for the mean phase shift and gets lower the more it differs from it. The solid line represents TE from neuronal pool 1 to pool 2 (forward), the dashed line from pool 2 to pool 1 (backward). Forward TE is clearly stronger than backward TE. 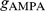/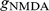 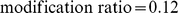, averaged over 100 trials.

**Figure 8:**
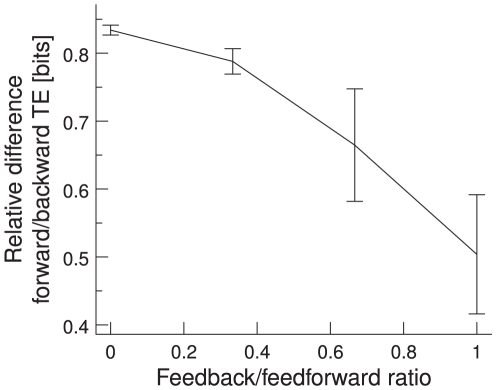
Relative differences in forward and backward TE. Differences in forward and backward TE are shown as a function of feedback/feedforward connection ratio, which is defined as 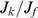. The difference between forward and backward TE becomes smaller as the feedforward and feedback connections become more similar. Error bars indicate 95% confidence intervals, 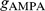/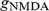 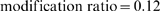, averaged over 100 trials.

We apply TE to the same data as in the previous section. However, we measure the TE between the MUA in the two pools and not only the spectral power at 60 Hz, as was done in the experiment. We find that the TE depends strongly on the phase relation in the gamma band between the spiking activities of the two groups of neurons. It is highest for the mean phase between the two signals and drops as it moves away from the mean. This is consistent with our results for correlation. The phase dependence is illustrated in Fig. 7. TE is plotted as a function of the mean phase shift. The solid line represents TE from the first to the second pool (forward) and the dashed line TE from the second to the first one (backward). Forward TE is stronger than backward TE, implying that TE correctly detects the causal dependence of the second neuronal pool on the first one. Forward TE is stronger than backward TE even if the feedforward and feedback connections are symmetrical (not shown). The stronger the feedforward and the weaker the feedback connections, the bigger the difference in the TE for the two directions, as shown in Fig. 8. We plot the relative difference in the TE, calculated as 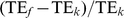. The feedback/feedforward ratio is defined as 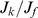. We use a feedback/feedforward ratio of 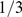 in the baseline simulations. To make sure the phase dependence is not only a by-product of changes in spectral power, we sorted the trials according to their spectral power in the gamma frequency band and calculated the phase dependence both for trials with power below and above the median. In both cases, the phase dependence is very similar (not shown).

### Different frequency bands

**Figure 9:**
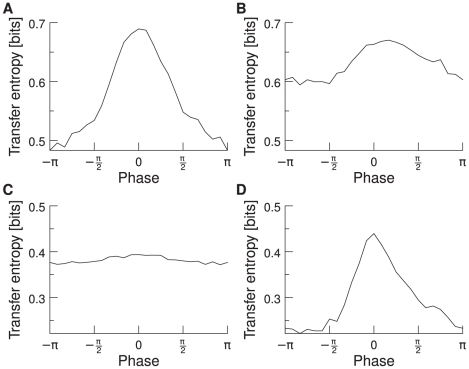
TE as a function of the mean phase shift in the gamma and beta band. The network oscillates strongly in one frequency band (either beta or gamma). The trials are sorted according to their phase shift either in the beta or gamma band. (**a**) Network oscillating in the gamma band. The trials are sorted according to their phase shift in the gamma band. (**b**) Network oscillating in the gamma band. The trials are sorted according to their phase shift in the beta band. (**c**) Network oscillating in the beta band. The trials are sorted according to their phase shift in the gamma band. (**d**) Network oscillating in the beta band. The trials are sorted according to their phase shift in the beta band. In all four graphics, 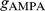/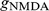 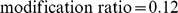, averaged over 100 trials.

Another result we obtain is that the phase dependence of information transport is not restricted to the gamma band. We find that even in simulations with a network oscillating strongly in the beta band (around 20 Hz), the TE is again highest for the mean phase shift. In Fig. 9, we compare the results for networks oscillating in the beta and gamma frequency band. Fig. 9a shows the TE for a network oscillating in the gamma band. The trials are sorted according to their phase relation in the gamma band. Fig. 9b shows the same network but with the trials now sorted according to their phase relation in the beta band. The phase dependence curve becomes a lot flatter and the optimal phase for maximal TE is much less pronounced. Fig. 9c shows the TE for a network oscillating in the beta band with trials sorted according to the phase relation in the gamma band. And Fig. 9d shows the TE for a network oscillating in the beta band with trials sorted according to the phase relation in the beta band. It becomes clear that it is the phase of the dominating frequency band that is responsible for high of low TE. We therefore conclude that it is not only the gamma band that has the ability to shape effective network connections via the phase, but that it is a general mechanism, observable in different frequency bands.

### Dependence of TE on gamma band power (without parameter modification)

**Figure 10:**
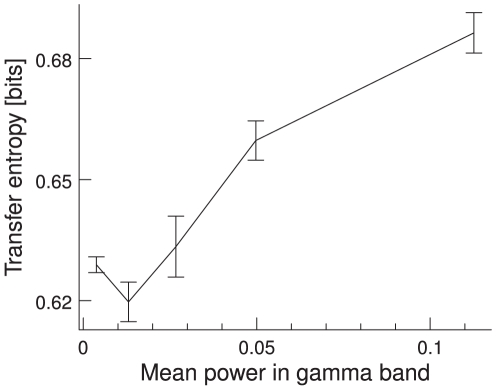
TE of trials sorted by power in the gamma band. Network parameters are kept fixed. The TE increases as a function of gamma power. 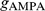/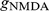 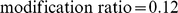, averaged over 100 trials. Error bars indicate 95% confidence intervals.

We further find that TE depends on the spectral power in the gamma band (30–85 Hz). For a fixed parameter set, we first sort all the trials according to their power in the gamma band into bins. In each of these bins, we measure the TE for the mean phase relation. The TE as a function of the power in the gamma band is plotted in Fig. 10. We find that the TE increases as a function of power. Note, however, that instead of sorting the trials according to their gamma band power for a fixed parameter set, we can also vary the parameters in the network. This allows us to vary the power over a wider range and the effect becomes clearer (see the next section).

### Dependence of TE on gamma band power (with parameter modification)

**Figure 11:**
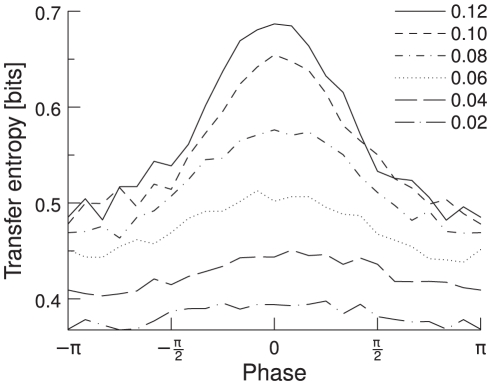
TE as a function of phase shifts and gamma oscillations. If the oscillations are strong in the gamma band (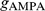/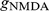 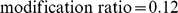), there is a clear phase shift between the two groups of neurons and the phase dependence curve is clearly bell shaped. If the oscillations are too low, there is no meaningful phase shift and the curve becomes flat (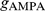/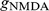 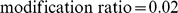). Averaged over 100 trials.

**Figure 12:**
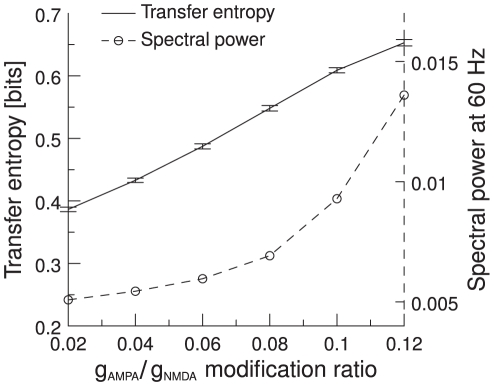
Mean TE as a function of gamma frequency band power. We plot the TE for six different 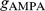/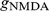 modification ratios (solid line). A higher 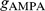/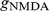 modification ratio causes the network to oscillate in the gamma frequency range and thus increases the power in the gamma frequency band (dashed line). Error bars indicate 95% confidence intervals; averaged over 100 trials.

In the previous section we have shown how TE depends on power in the gamma band for a fixed parameter set. Now we explicitly vary the amount of gamma power and study the TE dependence. Gamma band oscillations in a network of excitatory and inhibitory integrate-and-fire neurons appear when excitation is faster then inhibition [28]. Thus, we made the network oscillate by increasing AMPA conductance and decrease NMDA conductance. This change was applied to both excitatory and inhibitory neurons. In our simulations, we vary the 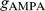/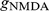 modification ratio from 0 to 0.12, which results in a gamma band that contains from 10 to 65% of power. If we sort the data according to its shift as in the previous section, we find that, for the different 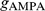/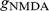 modification ratios, the TE shows a similar dependence on the phases. However, if the 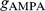/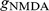 modification ratio is too low, the phase measurement is not reliable any more and the curve gets flat, consistent with the case of random phase distribution. Fig. 11 shows the TE as a function of phase shift for several different 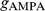/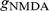 modification ratios. To summarize this result, we take the TE at the mean phase shift and plot it against the 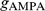/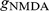 modification ratio. As the spectral power in the gamma band increases from 10 to 65%, the TE increases from 0.38 to 0.65 and thus shows strong positive correlation with the level of gamma band power (Fig. 12). In other words, the higher the gamma band synchronization between the two pools, the higher the information throughput. This result suggests that gamma band oscillations improve the signal processing in a network of IF neurons, as they increase the amount of transmitted information. This in turn confirms the idea that gamma band synchronization can shape effective networks, especially as it can influence the information transmission in a given direction, as shown above.

### Timing

**Figure 13:**
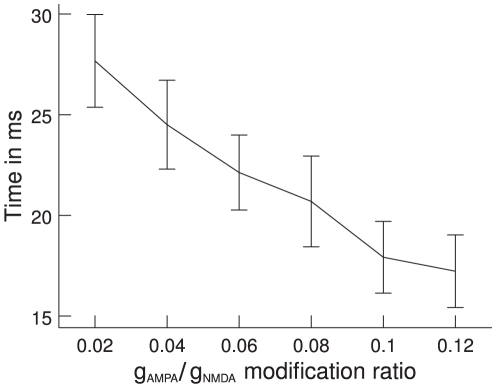
Rise times of TE as a function of gamma band power. Information starts flowing after stimulus onset when, consequently, TE starts rising. The plot shows the time required to reach 50% of the average TE. TE clearly rises faster for higher power in the gamma band (high 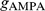/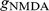 modification ratio). Error bars indicate 95% confidence intervals; averaged over 100 trials.

Finally, we are interested in whether the gamma band oscillations also have an influence on the speed of information exchange, on top of the increased amount of information exchange. To do this, we measure the time required until the stimulus presentation to the first pool leads to an increase in TE towards the second pool. We find that the onset of TE increase is significantly earlier when there is a lot of power in the gamma band. While for a 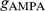/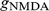 modification ratio of 0.02 it requires 28 ms to reach 50% of the average TE, for a 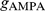/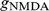 modification ratio of 0.12 it takes only 17 ms. The onset of information flow is clearly faster for higher levels of gamma band power (Fig. 13). This increase in speed is a further demonstration of how gamma oscillations increase network performance and shows how a network can be made more competitive.

## Discussion

### Communication through coherence

It has been hypothesized that interactions among neuronal groups depend on neuronal synchronization. Recent results show that gamma band oscillations and especially the phase relation in the gamma band can modify the strength of correlations in a network and therefore influence the effectiveness of connections in it [17]. These effects could be used as a mechanism to connect and disconnect areas in a network without altering the physical connections. Here, using a model network of IF neurons, we intend to test this hypothesis. We demonstrate that also in a model network, the correlation between two areas depends on the phase shift in the gamma band between these two areas. Our modeling approach enables us to use an information theoretical measure, as it allows us to generate as much data as needed for such a measure. Thus, we use transfer entropy, which has the advantage of being able to distinguish driving and responding elements in a network. We show that also for TE there is an optimal phase shift between two neuronal groups, where TE is highest. We study this phase dependence in different bands (beta and gamma). Our results demonstrate that, in a network with strong beta oscillations, TE depends on the phase shift in the beta band similarly to the way TE depends on the phase shift in the gamma band in a network with strong gamma oscillations. The ability to shape network connections seems therefore not to depend on the frequency range and seems to be a general mechanism. This confirms recent experimental results that have pointed out the importance of beta synchrony in functional integration [32], [33]. We also study how TE depends on power in a specific frequency band. We do this here for the gamma band. For a fix set of parameters, we sort the trials in a simulation according to their power in the gamma band. We find that within a simulation, the trials with high gamma power have a high TE. Then we modify the parameters and vary the gamma power over a wider range. Again, we find that TE increases as a function of gamma power. Finally, we reveal that it is not only the amount of exchanged information that increases but also the speed: The higher the power in the gamma band, the earlier the onset of the information flow.

Our results support the CTC hypothesis. If the effective connections in a network are to be influenced by the phase lock in a specific frequency band between two areas, it is important that it not only affects the coherence between them, but also the throughput of information in a specific direction. By measuring TE instead of the Spearman rank coefficient, we extend the work of [17]. Our result is also more general, as we use the rates to measure TE and not only the 60 Hz power. Our study of different frequency bands is a further extension. We provide evidence that the CTC mechanism is not restricted to the gamma band, but also functions in different frequency bands. In addition, our modeling approach also enables us to study how the information transport depends on the total power in a specific frequency band. Our finding that TE increases as a function of power suggests that both the phase and the power in a specific frequency band are important to shape effective connections in a network. The phase dependence of information transmission is not only a byproduct of the power dependence, as we find the same phase dependence both in trials with high gamma power as well as in trials with low gamma power. Furthermore, we demonstrate that the onset of information exchange depends on the power, which contributes to effectively shaping the connections in a network. We have already shown in the context of attention that gamma power increases the network effect of an attentional bias and that it makes the network more efficient [34]. Here, we can confirm this finding and put it in a more general context, independent of attention.

### Implications for visual information transmission

As we are modeling results from visual cortical areas, we can assume that the neuronal clusters in the model transmit largely visual information. Several recent studies have contributed to the understanding of visual information transmission. These studies suggest that LFP power gradually increases as a function of stimulus contrast and gamma band LFP power increases differentially, that is, to a higher extent with respect to the baseline than relative to either higher or lower bands [35]. For the highest stimulus contrast, these authors report a clear peak in the gamma frequency band. In other words, the contrast dependence of the LFP is different in different frequency bands and the LFP power spectrum changes shape depending on contrast, with a peak in the gamma band emerging at high contrast. [36] studied the encoding of naturalistic sensory stimuli in LFPs and spikes. They found that the most informative LFP frequency ranges were 1–8 Hz and 60–100 Hz. They showed that the LFP in the 60–100 Hz high gamma band showed little noise correlation during visual stimulation but showed the highest observed signal correlation across all LFP frequencies. The high gamma band also had the highest proportional power increase during visual stimulation. These experimental results are supported by the modeling work of [37]. These authors showed that their modeling network encoded static input spike rates into gamma-range oscillations generated by inhibitory-excitatory neural interactions. In sum, these reports indicate that the gamma frequency range is the one used most frequently to encode visual information in the visual cortex and that visual information is encoded by the power of gamma range oscillations. These observations, together with our result that gamma oscillations increase both the overall amount and the speed of information indicates that information about preferred stimuli is treated preferentially and, in consequence, that cortical modules mostly exchange information about their preferred stimuli.

In sum, we provide results to support the CTC hypothesis, we show evidence that CTC is a general mechanism independent of a specific frequency band and show that not only the phase but also the power is important to effectively shape the flow of information in a network.
